# Overcoming chemo/radio-resistance of pancreatic cancer by inhibiting STAT3 signaling

**DOI:** 10.18632/oncotarget.7336

**Published:** 2016-02-12

**Authors:** Xiaoqing Wu, Wenhua Tang, Rebecca T. Marquez, Ke Li, Chad A. Highfill, Fengtian He, Jiqin Lian, Jiayuh Lin, James R. Fuchs, Min Ji, Ling Li, Liang Xu

**Affiliations:** ^1^ Departments of Molecular Biosciences and Radiation Oncology, University of Kansas, Lawrence, KS, USA; ^2^ Department of Radiation Oncology, University of Michigan Medical School, Ann Arbor, MI, USA; ^3^ School of Chemistry and Chemical Engineering, Southeast University, Nanjing, Jiangsu, China; ^4^ Department of Biochemistry and Molecular Biology, Third Military Medical University, Chongqing, China; ^5^ Department of Pediatrics, College of Medicine, Ohio State University, Columbus, OH, USA; ^6^ Division of Medicinal Chemistry and Pharmacognosy, College of Pharmacy, Ohio State University, Columbus, OH, USA; ^7^ Department of Cell Biology and Cell Engineering Research Centre, State Key Laboratory of Cancer Biology, Fourth Military Medical University, Xi'an, Shanxi, China

**Keywords:** chemo/radio-resistance, pSTAT3, pancreatic cancer, lip-FLLL32, CSCs

## Abstract

Chemo/radio-therapy resistance to the deadly pancreatic cancer is mainly due to the failure to kill pancreatic cancer stem cells (CSCs). Signal transducer and activator of transcription 3 (STAT3) is activated in pancreatic CSCs and, therefore, may be a valid target for overcoming therapeutic resistance. Here we investigated the potential of STAT3 inhibition in sensitizing pancreatic cancer to chemo/radio-therapy. We found that the levels of nuclear pSTAT3 in pancreatic cancer correlated with advanced tumor grade and poor patient outcome. Liposomal delivery of a STAT3 inhibitor FLLL32 (Lip-FLLL32) inhibited STAT3 phosphorylation and STAT3 target genes in pancreatic cancer cells and tumors. Consequently, Lip-FLLL32 suppressed pancreatic cancer cell growth, and exhibited synergetic effects with gemcitabine and radiation treatment *in vitro* and *in vivo*. Furthermore, Lip-FLLL32 reduced ALDH1-positive CSC population and modulated several potential stem cell markers. These results demonstrate that Lip-FLLL32 suppresses pancreatic tumor growth and sensitizes pancreatic cancer cells to radiotherapy through inhibition of CSCs in a STAT3-dependent manner. By targeting pancreatic CSCs, Lip-FLLL32 provides a novel strategy for pancreatic cancer therapy via overcoming radioresistance.

## INTRODUCTION

Pancreatic cancer is estimated to responsible for 40,560 deaths and 48,960 newly diagnosed cases in the United States in 2015 [1]. Most patients die within the first year of their diagnosis, setting the five-year survival rate to only 6% [2]. Surgery offers the only option to cure patients with pancreatic cancer, however, only 20% of patients are eligible for surgery [3, 4]. Systemic chemotherapy or chemoradiotherapy only has a modest benefit on most patients with advanced disease. Both treatments have high frequency of relapse and patients develop resistant eventually [4, 5]. The failure to kill tumor-initiating cells or cancer stem cells (CSCs) by conventional chemo/radio-therapy is one of the major reasons for recurrence and resistance [6, 7]. Targeting molecules and signaling pathways associated with CSCs is one of potential strategies to overcome these problems.

Recent studies demonstrate that signal transducer and activator of transcription 3 (STAT3) plays critical roles in initiation and progression of pancreatic cancer, especially in mutant *KRAS*-mediated pancreatic ductal adenocarcinomas (PDAC) [8–10]. In response to cytokines or growth factors, activated STAT3 functions as a nuclear transcription factor by regulating genes involved in proliferation, survival, angiogenesis and invasion, as well as genes encoding key cancer-promoting inflammatory mediators [11, 12]. More importantly, STAT3 signaling has central roles in CSC maintenance [11, 13, 14] and therapeutic resistance [15, 16]. We have shown that blocking upstream activators of STAT3 inhibits pancreatic tumor growth and post-radiation recurrence partly by eradicating CSCs [17, 18]. Since STAT3 serves as a point of convergence for numerous oncogenic signaling pathways, directly targeting STAT3 might be a more effective strategy for pancreatic cancer treatment, especially for overcoming chemo/radio-therapy resistance that derives from CSCs.

Inhibiting intermolecular interactions between STAT3 and upstream proteins by targeting STAT3 Src homology-2 (SH2) domain is one of strategies that directly block STAT3 activity [19]. FLLL32, an analogue of Curcumin, was designed to selectively bind to STAT3 SH2 domain, thereby inhibiting STAT3 phosphorylation and DNA-binding activity [20]. FLLL32 potently and specifically inhibited STAT3 (no inhibition of STAT1) and exhibited growth-repressive activity in cancer cells with constitutively activated STAT3 [20–22]. However, the delivery of FLLL32 has been limited by its low aqueous solubility, which is the same as its parent compound. Many *in vivo* studies introduced intraperitoneal injection of FLLL32 dissolved in Dimethyl sulfoxide (DMSO) or Cremophor EL/ethanol formulation [20, 23, 24]. These are compromising formulations due to the fact that high dose of DMSO is toxic and Cremophor is associated with serious side effects of hypersensitivity, nephrotoxicity and neurotoxicity [25, 26]. Moreover, drug efficacy is lower when administrated by intraperitoneal injection than by intravenous (i.v.) injection. Thus, in this study, we prepared PEGylated liposomal FLLL32 that allowed for i.v. administration. In addition to its enhanced biocompatibility and reduced toxicity, PEGylated liposomes with size around 100 nM can be passively delivered into solid tumors via the “enhanced permeability and retention (EPR)” effect [27, 28] and escape the reticulo-endothelial system (RES) clearance with the PEG shielding effect [29].

Here, we show for the first time that liposomal delivery of FLLL32, a STAT3 phosphorylation inhibitor, efficiently suppressed pancreatic cancer xenograft tumor growth, and sensitized pancreatic cancer cells to radiotherapy *in vitro* and *in vivo* by inhibiting STAT3 signaling in CSCs potentially.

## RESULTS

### Increased pSTAT3 expression in human pancreatic adenocarcinoma is associated with poor clinical outcome

**Figure 1:**
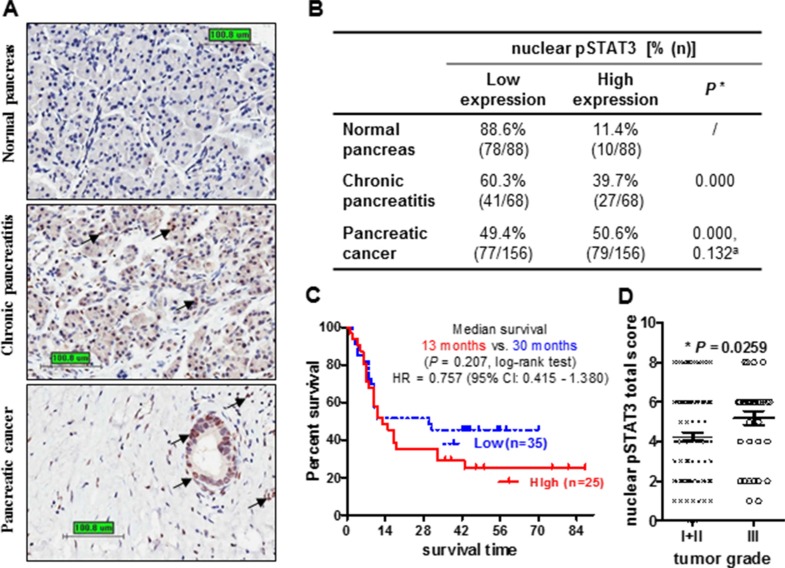
Increased nuclear pSTAT3 expression in human pancreatic adenocarcinoma is associated with poor clinical outcome (**A**) Representative images of pSTAT3 immunohistochemistry staining in pancreatic tissues (Bar: 100.8 μm. Magnification: 400×). Black arrows highlight positive staining. (**B**) Percentage of nuclear pSTAT3 expression in non-neoplastic tissue and PDCA. *The percentage of high nuclear pSTAT3 in chronic pancreatitis and pancreatic cancer was compared to that of normal pancreas. ^a^The percentage of high nuclear pSTAT3 in pancreatic cancer was compared to that of chronic pancreatitis. Chi-square test. (**C**) Kaplan-Meier analysis of the overall survival of 60 patients comparing high and low nuclear pSTAT3. Patients with high pSTAT3 have a shorter medium survival comparing to those with low pSTAT3 (13 months vs 30 months, *P* = 0.207, log-rank test). (**D**) Nuclear pSTAT3 expression in pancreatic tissues with low grade or high grade. Patients with high grade tumors (III, *n* = 37) have higher nuclear pSTAT3 expression compared to those with low grade tumors (I + II, *n* = 119) (*p* = 0.0259, *t*-test).

To explore the clinic-pathological significance of pSTAT3 in pancreatic cancer and the utility of STAT3 inhibition in sensitizing pancreatic cancer to chemo/radio-therapy, we first measured pSTAT3 expression by immunohistochemistry in 156 pancreatic cancer samples paired with normal tissues resected from primary pancreatic tumors and adjacent non-tumor areas. Nuclear pSTAT3 was negative to weakly expressed (defined as low expression) in normal pancreas (88.6%) and chronic pancreatitis (60.3%), while was expressed moderately to strongly (defined as high expression) in PDAC (50.6%) (Figure 1A and 1B). The ratio of high nuclear pSTAT3 expression in PDAC was significantly higher than that of in normal pancreas (50.6% vs 11.4%).

We next investigated the correlation between nuclear pSTAT3 expression and clinic-pathological parameters. To begin with, we examined the association of nuclear pSTAT3 expression with survival status of 60 pancreatic cancer patients that had available survival data by Kaplan-Meier survival analysis. Patients with high pSTAT3 expression had a shorter median survival time than patients with low pSTAT3 expression (13 months *vs*. 30 months, *P* = 0.207, log-rank test, Figure 1C), though did not reach statistical significance. The 5-year survival rate for patients whose tumors expressed either high or low levels of pSTAT3 was of 28% and 44%, respectively. However, high expression of nuclear pSTAT3 was significantly correlated with high tumor grade (*P* = 0.0259) and glandular cancer (*P* = 0.037) (Figure 1D, Supplementary Table 1). No significant correlation exists in age, gender, tumor size and location, TNM stage, AJCC stage, smoking, drinking as well as patient survival. Taken together, increased nuclear pSTAT3 staining correlates with advanced tumor grade and poor patient outcome. Therefore, targeting STAT3 by small molecule inhibitor FLLL32 could be a potential therapeutic strategy for inhibiting pancreatic cancer progression and overcoming chemo/radio-resistance.

### Liposomal FLLL32 is effectively and specifically delivered into pancreatic tumors

**Figure 2:**
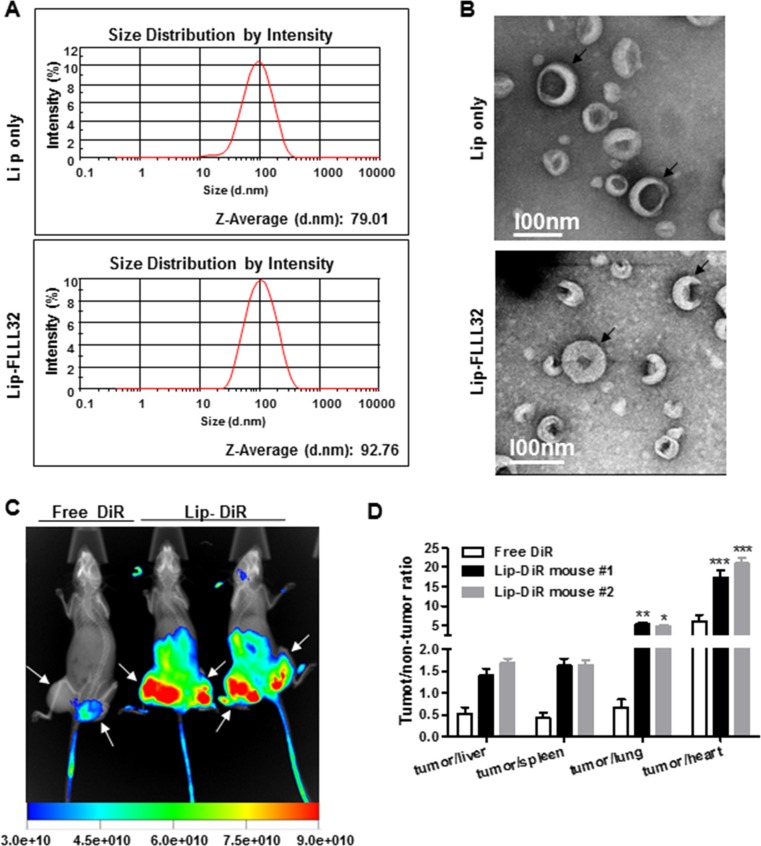
Lip-FLLL32 is effectively delivered to pancreatic tumors (**A**) Representative size distributions of empty liposome (up) and Lip-FLLL32 (down) measured by Malvern Nano-ZS90. (**B**) Representative STEM images of empty liposome (up) and Lip-FLLL32 (down). Black arrows highlight the typical liposomes. Biodistribution study: (**C**) NIR and X-ray overlaid image of mice 24 hours after injection of free DiR or liposomal DiR and FLLL32. White arrows highlight the tumors. Intensity bar is shown at the bottom. (**D**) The tumor/non-tumor ratios of fluorescence intensity in mice 96 hours after injection of free DiR or liposomal DiR and FLLL32 (*P < 0.05, **P < 0.01, ***P < 0.001, two-way ANOVA, *n* = 2).

To improve *in vivo* delivery of FLLL32, we prepared liposomes encapsulating FLLL32 (Lip-FLLL32) by thin-film hydration method. Empty liposomes (Lip only) were also prepared as a vehicle control. The sizes of Lip only and Lip-FLLL32 were 78.92 ± 5.54 nm (*n* = 3) and 92.29 ± 8.19 nm (*n* = 3), respectively, measured by dynamic laser scattering. This size distribution of Lip-FLLL32 indicates that it could passively target solid tumors via increased permeable tumor vasculature known as the “enhanced permeability and retention (EPR)” effect, as it is generally assumed that particles less than 200 nm in diameter are able to extravasate to the tumor site [27]. Representative size distributions of both were shown in Figure 2A. By scanning transmission electron microscopy (STEM), we found that the surface of Lip-FLLL32 was bumpy, indicative of successful embedding of FLLL32 into the liposome, while the surface of Lip only was smooth (Figure 2B).

Next, we investigated the tumor-targeting efficiency and biodistribution of Lip-FLLL32 in the PANC-1 xenograft mouse model. We encapsulated FLLL32 into the liposomes together with DiR, a lipophilic NIR fluorescence dye, which can be used to track the delivery of the liposomes *in vivo*. The liposomes were administered by tail vein injection into nude mice containing PANC-1 xenografts. The fluorescence signals were determined 24 h after injection. The xenografts in the mice injected with liposomal DiR plus FLLL32 had significantly stronger fluorescence signals than that of in the mouse injected with free DiR, whose fluorescence signals were limited to the tail but not to the tumors (Figure 2C). This result indicated that the liposomal FLLL32 was specifically delivered to the xenografts. We further compared the fluorescence intensity of tumors with that of other organs. As shown in Figure 2D, the fluorescence intensity ratios of tumor to heart or lung in the mice injected with liposomal DiR plus FLLL32 were 3.1 or 7.1 folds of that in mouse injected with free DiR (19.2 ± 2.6 *vs*. 6.1 ± 2.3, *P* < 0.001 for heart, and 5.0 ± 0.3 *vs*. 0.7 ± 0.3, *P* < 0.01 for lung, respectively.). As for liver and spleen, the two main internal organs that are involved in nonspecific clearance of nanoparticles through their reticuloendothelial system (RES), the tumor/liver or tumor/spleen ratios in liposomal DiR and FLLL32 treated mice were 1.5 ± 0.2 or 1.6 ± 0.01, which were 2.9 or 3.8 folds of that in the mouse injected with free DiR, respectively. In addition, the fluorescent intensity of tumors given liposomal DiR and FLLL32 was about 4-fold higher than that of tumors received free DiR (Figure S1). All these data reveal that Lip-FLLL32 was effectively and specifically accumulated in tumors after systemic administration *in vivo*.

### Lip-FLLL32 dose-dependently inhibits STAT3 phosphorylation and transcriptional activity

**Figure 3:**
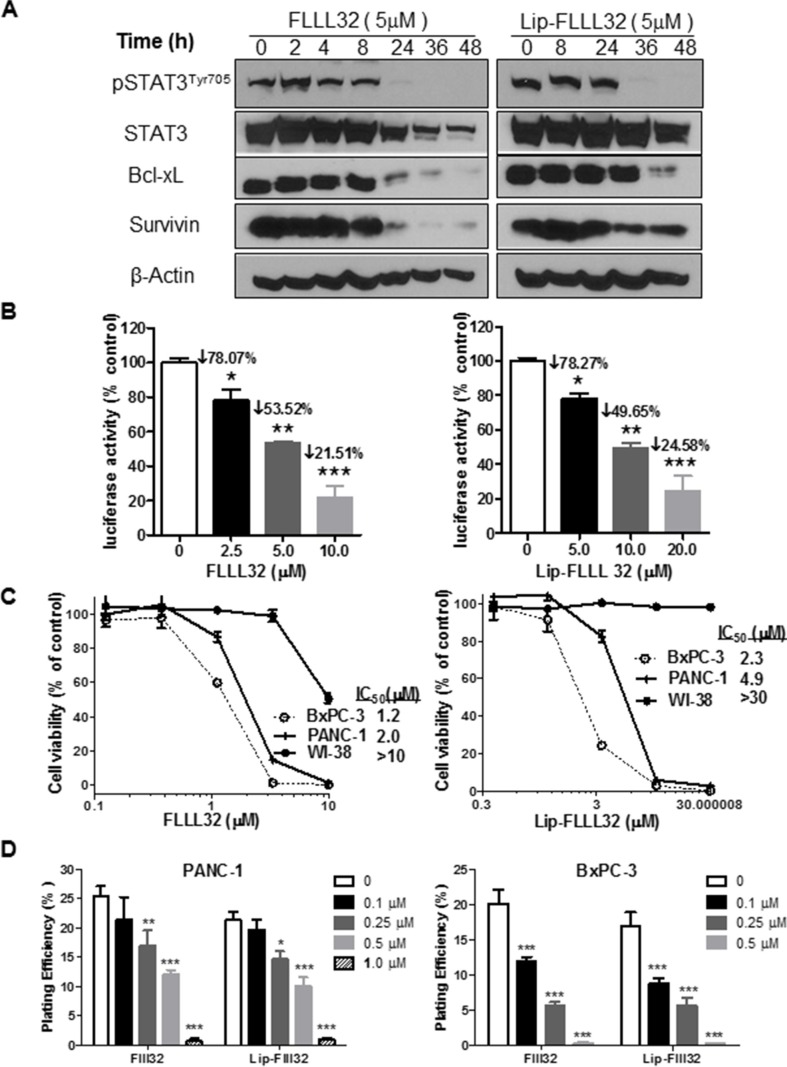
Lip-FLLL32 inhibits pancreatic cancer cell growth *in vitro* in a STAT3 dependent way (**A**) Western blot analysis of total STAT3, pSTAT3 and its target proteins in PANC-1 cells treated with 5 μM FLLL32 or Lip-FLLL32 at indicated time points. β-Actin was used as loading control. (**B**) STAT3-dependent transcriptional luciferase activity assay in PANC-1 cells treated with FLLL32 or Lip-FLLL32. PANC-1 cells were co-transfected with pLucTKS3 luciferase reporter construct and beta-galactosidase plasmid, and then treated with FLLL32 or Lip-FLLL32 for 24 hours. Values are mean ± SD from two independent experiments. *P < 0.05, **P < 0.01, ***P < 0.001, one-way ANOVA. (**C**) The cytotoxicity of free FLLL32 (left) and Lip-FLLL32 (right) against BxPC-3, PANC-1 and WI-38 cell lines. (**D**) Colony formation assay in PANC-1 (left) and BxPC-3 (right) cells treated with free FLLL32 or lip-FLLL32 at indicated doses. DMSO was used as vehicle control of FLLL32 and Lip only was used as vehicle control of Lip-FLLL32. *P < 0.05, **P < 0.01, ***P < 0.001, two-way ANOVA.

To validate whether Lip-FLLL32 effectively inhibits STAT3 phosphorylation and its transcriptional activity as FLLL-32 does, we firstly screened the optimal inhibitory concentration of FLLL32 for PANC-1 and BxPC-3 cells, which have constitutively activated STAT3. As shown in Figure S2A, FLLL32 dose-dependently down-regulated the protein levels of pSTAT3 and its target Cyclin D1, Survivin and Bcl-xL, whereas increased cleaved PARP, with totally blockage of pSTAT3 at the concentration of 5 μM for PANC-1 and 1 μM for BxPC-3, respectively. We then determined the effect of Lip-FLLL32 on STAT3 phosphorylation and its corresponding target genes expression by 5 μM at different time points for PANC-1. Similar to FLLL32, Lip-FLLL32 reduced the protein level of pSTAT3, Bcl-xL and Survivin in a time-dependent manner (Figure 3A). However, the time point of totally blockage of pSTAT3 and its target proteins in Lip-FLLL32 treated cells occurred 12 h later than in free FLLL32 treated cells, which might be due to the slow release of FLLL32 from the liposomes. Furthermore, we measured pSTAT3 transcriptional activity using luciferase assay as described previously [20]. We found both FLLL32 and Lip-FLLL32 dose-dependently decreased pSTAT3 transcriptional activity, although Lip-FLLL32 required a two-fold higher dose than that of FLLL32 to achieve the same inhibitory effect, which might be also due to the slower release feature of Lip-FLLL32 (Figure 3B). Therefore, these data indicate that Lip-FLLL32 is functionally effective in inhibiting pSTAT3 signaling, although it required relatively longer time as compared to free FLLL32. Nevertheless, the delayed inhibition of pSTAT3 pathway may provide an advantage of Lip-FLLL32 in long-term administration.

### Lip-FLLL32 effectively inhibits pancreatic cancer cell growth *in vitro and in vivo*

To further determine whether Lip-FLLL32 inhibits cell growth *in vitro* as FLLL32 does, we carried out the MTT-based cytotoxicity assay of Lip-FLLL32 against a panel of pancreatic cancer cell lines as well as an immortalized human lung fibroblast cell line WI-38. We found that both FLLL32 and Lip-FLLL32 dose-dependently inhibited cell growth in all the pancreatic cancer cell lines tested, but not in WI-38 cells which have low level of pSTAT3 (Figure 3C & Figure S2B). The IC_50_ values of Lip-FLLL32 for all detected cell lines were approximately two-fold as that of FLLL32, consistent with their inhibitory effects on STAT3 phosphorylation and transcriptional activity. But in colony formation assays which were carried out as described previously [30, 31], both FLLL32 and Lip-FLLL32 dose-dependently decreased the plating efficiency in PANC-1 and BxPC-3 cells at the equal dose and the equal time (Figure 3D). Different from the cytotoxicity assay and Western blot based pSTAT3 detection that treatment lasted less than 96 hours, colony formation assay required longer time (usually up to two weeks) of treatment, in which time frame encapsulated FLLL32 might be completely released from the liposomes. Thus, the same extent of inhibitory effects by Lip-FLLL32 and FLLL32 were detected.

**Figure 4:**
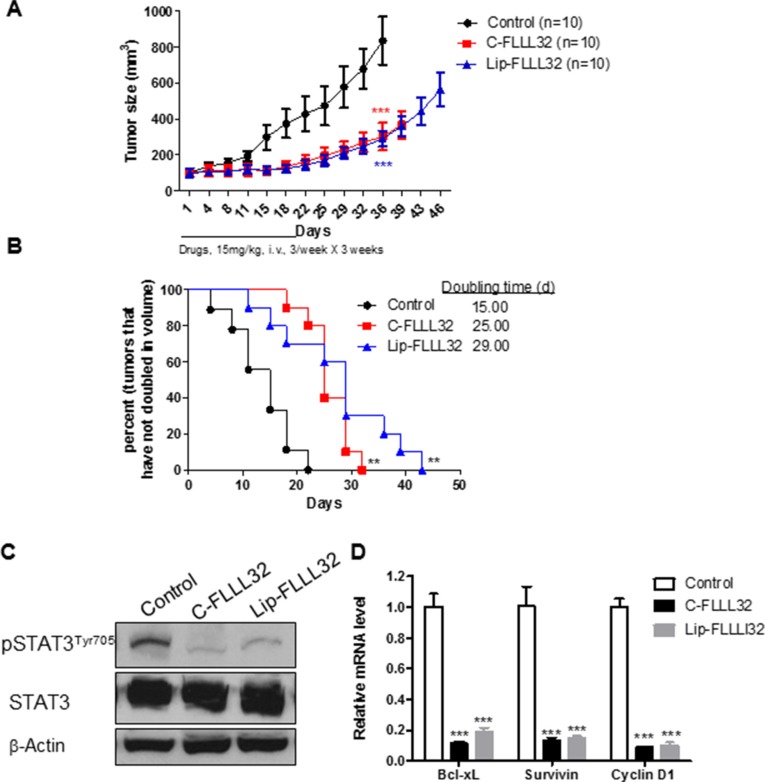
Lip-FLLL32 inhibits pancreatic cancer xenograft growth *in vivo* in a STAT3 dependent way Nude mice bearing PANC-1 xenografts were intravenously injected with 15 mg/kg Cremophor formulated FLLL32 or 15 mg/kg liposome formulated FLLL32 three times a week for three weeks or left untreated. (**A**) Tumor growth curves of mice in three groups were plotted up to Day 46. The data are mean ± SEM (*n* = 10). ***P < 0.001, two-way ANOVA, by comparing C-FLLL32 or Lip-FLLL32 treated group with untreated control group at day 36. (**B**) Kaplan-Meier analysis of tumor size doubling time of mice in three groups. The medium doubling time for control, C-FLL32 and Lip-FLLL32 is 15, 25 and 29, respectively. **P < 0.01, log-rank test, by comparing two treated groups with untreated control group. (**C**) Phosphorylation and total STAT3 protein expression levels were analyzed in representative tumor samples from untreated control, C-FLLL32 and Lip-FLLL32 treated groups. (**D**) qRT-PCR analysis of relative mRNA levels of STAT3 target genes in xenograft tumor samples. ***P < 0.001, two-way ANOVA, by comparing C-FLLL32 or Lip-FLLL32 treated sample with untreated control sample.

Next, we examined whether Lip-FLLL32 had safely therapeutic effects *in vivo*, which require efficient drug delivery during long-term administration. To begin with, we first determined the maximal tolerated dose (MTD) of Lip-FLLL32 in mice, with Cremophor formulated FLLL32 (C-FLLL32) as a solvent control. As shown in Supplementary Table 2, the MTD for liposomal FLLL32 is over 22.5 mg/kg but for C-FLLL32 is only 15 mg/kg, indicating liposomal FLLL32 is more tolerable in mice. Based on this data, 15 mg/kg Lip-FLLL32 or C-FLLL32 was chosen for *in vivo* efficacy studies. When administrated alone, both Lip-FLLL32 and C-FLLL32 significantly inhibited tumor growth (*P* < 0.001 versus untreated control group for both formulations, *n* = 10, Figure 4A), and prolonged the tumor-doubling time correspondingly (*P* < 0.01 versus untreated group for both formulations, *n* = 10, Figure 4B). There was no statistical difference between C-FLLL32 and Lip-FLLL32 in reducing tumor growth. The median tumor doubling time was 15, 25, and 29 days for the control group, C-FLLL32 group and Lip-FLLL32 group respectively, indicating that Lip-FLLL32 more efficiently delayed tumor growth than C-FLLL32 at the same dosage although there was no significant difference between the two in the tumor-doubling time curves. During the experimental period, the bodyweight of the control mice and two FLLL32-treated mice all changed within 10%, thus both C-FLLL32 and Lip-FLLL32 at the dose of 15 mg/kg of FLLL32 were well tolerated in mice (Figure S3A).

To determine whether the *in vivo* tumor growth inhibitory effect of Lip-FLLL32 was connected to the reduction of STAT3 phosphorylation and transcriptional activity, one tumor from each group after one-week's treatment was removed to detect the expression of pSTAT3 and its target genes. We found that the pSTAT3 protein levels in both C-FLLL32 and Lip-FLLL32 treated tumors were greatly down-regulated (Figure 4C). Meanwhile, qRT-PCR analysis revealed that the relative mRNA levels of STAT3 target genes *Bcl-xL*, *Survivin* and *CCND1* were all sharply decreased by 80% in two treated tumors, as compared to the untreated control (*P* < 0.001 for both treatments, Figure 4D). These results suggested that FLLL32 at the dose of 15 mg/kg delivered by the two formulations inhibited STAT3 phosphorylation and transcriptional activity at the similar extent *in vivo*. To investigate how Lip-FLLL32 decreased tumor growth, tumor tissues at the end of treatment were collected and processed for histologic examination of apoptosis using TUNEL staining and angiogenesis using CD31 immunostaining. Both C-FLLL32 and Lip-FLLL32 treatment significantly increased TUNEL-positive tumor cells and reduced CD31-postitive endothelial cells than untreated control (Figure S3B–S3C). This result demonstrates that Lip-FLLL32 inhibited tumor growth via inducing tumor cell apoptosis and reducing tumor angiogenesis. This is consistent with the fact that STAT3 downstream targets are involved in anti-apoptotic and angiogenesis pathways. Taken together, Lip-FLLL32 efficiently and safely delayed *in vivo* pancreatic tumor growth in a STAT3-dependent manner.

### Lip-FLLL32 sensitized pancreatic cancer to chemo/radio-therapy

**Figure 5:**
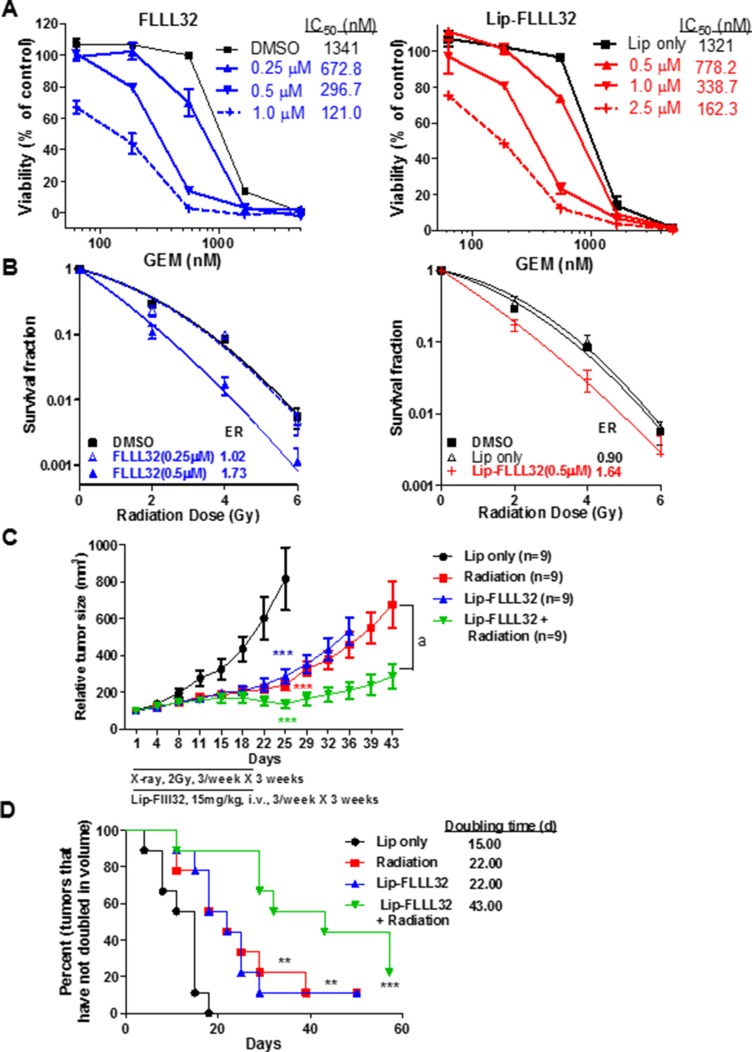
Lip-FLLL32 sensitizes pancreatic cancer to chemo/radiotherapy (**A**) Cytotoxicity of Gemcitabine combination with free FLLL32 (left) or Lip-FLLL32 (right) against PANC-1 cells. (**B**) Clonogenic survival assay of PANC-1 cells treated by FLLL32, radiotherapy or their combination. Survival fractions were plotted, and the enhancement ratios (ER) by FLLL32 or Lip-FLLL32 were calculated *vs*. DMSO. (**C**) PANC-1 xenograft nude mice were treated with empty liposome, 2 Gy radiation, 15 mg/kg Lip-FLLL32 or combination three times a week for three weeks. Tumor growth curves of mice were plotted up to Day 43. The data are mean ± SEM (*n* = 9). ***P < 0.001, two-way ANOVA, by comparing Radiation, Lip-FLLL32 or combination treated group with Lip only group at day 25; a, *P* < 0.001, two-way ANOVA, by comparing combination group with Radiation group. (**D**) Kaplan-Meier analysis of tumor size doubling time of mice in four groups. The medium doubling time for Lip only, Radiation, Lip-FLLL32 and combination treated groups is 15, 22, 22 and 43, respectively. **P < 0.01, ***P < 0.001, log-rank test, by comparing three other groups with untreated control group.

In pancreatic cancer, neither chemotherapy nor radiotherapy alone is able to effectively block cancer progression and both treatments develop resistance eventually [4, 32]. Besides, indirect inhibition of STAT3 phosphorylation/activation alone using small molecules that antagonize STAT3 upstream growth factor and cytokine receptor only shows modest efficacy [19]. We thus turned to explore whether directly targeting STAT3 with Lip-FLLL32 could enhance the inhibitory efficacy of gemcitabine or radiation on pancreatic cancer tumor growth. We first examined the sensitivity alteration of PANC-1 cells to gemcitabine with or without combined STAT3 inhibition. As shown in Figure 5A, with the doses of free FLLL32 or Lip-FLLL32 increased, the IC_50_ of gemcitabine decreased correspondingly, suggesting that both free FLLL32 and Lip-FLLL32 sensitized PANC-1 cells to gemcitabine (Figure 5A). In comparison to free FLLL32, Lip-FLLL32 exhibited similar inhibition at the higher dose, potentially due to its slow release during this short time period of 96 hours. However, in the clonogenic assays which were carried out as described previously [31] and lasted for two weeks, both free FLLL32 and Lip-FLLL32 enhanced radiation-induced clonogenic cell death in the same degree with a radiation dose enhancement ratio (ER) of 1.73 and 1.64, respectively (Figure 5B). Since the ER values are larger than 1.20 [33], both free FLLL32 and Lip-FLLL32 are considered to be able to radiosensitize pancreatic cancer. These *in vitro* results demonstrate that liposomal FLLL32 potentially sensitize patients to chemotherapy or radiotherapy.

We next examined the radiosensitization potential of Lip-FLLL32 *in vivo* in PANC-1 xenograft mouse model. As shown in Figure 5C, X-ray radiation or Lip-FLLL32 alone significantly inhibited tumor growth (*P* < 0.001 versus control group for both groups, *n* = 9). But when radiation was combined with Lip-FLLL32, tumors were shrunk significantly as compared to radiation alone (*P* < 0.001). Doubling time of tumors in each group was shown in Figure 5D. Either radiation or liposomal FLLL32 treatment alone delayed tumor doubling time by 7 days as compared to Lip only control group (*P* < 0.01, *n* = 9), whereas the combination of liposomal FLLL32 with radiation delayed tumor doubling time for 28 days (*P* < 0.001, *n* = 9). The bodyweight of the mice in four groups all changed within 10% (Data not shown). Together, our *in vitro* and *in vivo* data demonstrate that Lip-FLLL32 sensitizes PANC-1 xenografts to radiotherapy.

### Lip-FLLL32 sensitized pancreatic cancer to chemo/radio-therapy by reducing cancer stem cells

**Figure 6:**
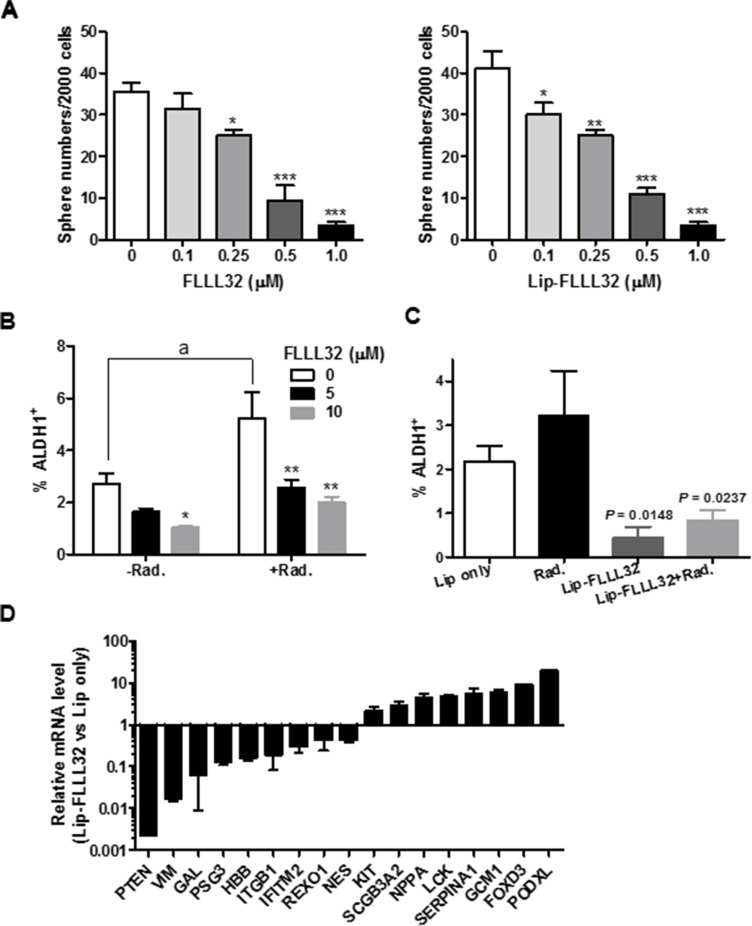
Lip-FLLL32 sensitizes pancreatic cancer to radiotherapy by reducing pancreatic CSCs (**A**) Tumorsphere formation assay of free FLLL32 (left) or liposomal FLLL32 (right) treated PANC-1cells at indicated doses. Spheres of each treatment were counted and compared to vehicle controls. *P < 0.05, **P < 0.01, ***P < 0.001, one-way ANOVA. (**B**) Flow cytometry analysis of ALDH1^+^ population in PANC-1 cells treated with FLLL32 at indicated doses for 24 hours combining with or without 10 Gy radiation treatment. *P < 0.05, **P < 0.01, two-way ANOVA; a, *P* < 0.01, *t*-test, Radiation only vs no treatment. (**C**) Flow cytometry analysis of ALDH1^+^ population in xenograft tumor samples from mice treated with Lip only, radiation, liposomal FLLL32, or their combination for one week. (**D**) The relative mRNA expression of stem cell-associated genes in Lip-FLLL32 treated PANC-1 cells was analyzed using Human Stem Cell Primer Library and quantitative real-time reverse transcription PCR. Gene expression in Lip-FLLL32 treated cells was normalized to β-actin and β-2-microglobulin and set relative to that of Lip only treated PANC-1 cells.

Emerging evidence suggests that the persistence of CSCs might be the cause of the high frequency of relapse and failure of current cancer therapies [34–36]. Since STAT3 signaling has central roles in CSCs [37–39], our above effective combination treatment might be resulted from both eliminating the proliferating cancer cells by radiation and eradicating CSCs by Lip-FLLL32. To test this hypothesis, we investigated whether the Lip-FLLL32 mediated suppression of pancreatic cancer cell growth is associated with reduction of CSCs and inhibition of their function. We first examined the influence of Lip-FLLL32 alone on *in vitro* tumorigenic capability of CSCs by tumorsphere culture. As shown in Figure 6A, both free FLLL32 and Lip-FLLL32 dose-dependently decreased sphere numbers, indicating that FLLL32 can reduce the tumorsphere-forming cell population in BxPC-3 cells with the doses tested in this assay. Aldehyde dehydrogenase 1 (ALDH1) is a widely accepted marker for identifying CSCs [40, 41], especially for marking pancreatic cancer cells that have stem cell and mesenchymal features [42]. We then determined the effect of FLLL32 combined with or without radiotherapy on ALDH1 positive cells using ALDEFLUOR assay. PANC-1 cells were treated with FLLL32 with or without 10 Gy radiation and harvested 24 hours later for the assay. 10 μM FLLL32 reduced the ALDH1^+^ cell population in PANC-1 cells (*P* < 0.05), and abrogated the radiation-induced increase of ALDH1^+^ cell population (*P* < 0.01) (Figure 6B). Attenuation of radiation-induced ALDH1^+^ cell population increase was also observed at a lower concentration of 5 μM. We also examined the ALDH1^+^ cell population in PANC-1 xenografts received the combination therapy (Figure 6C and S4). Compared to empty liposomes, Lip-FLLL32 alone significantly inhibited ALDH1^+^ cell population in pancreatic cancer cells (*P* = 0.0148). Lip-FLLL32 abolished the radiation-induced increase of ALDH1^+^ population to a level lower than that of Lip-only control (*P* = 0.0237). The data validate that the above effective combination treatment actually eliminated both CSCs and proliferating cancer cells. We further screened the Human Stem Cell Primer Library to explore other potential stem cell genes that would be affected by Lip-FLLL32 (Figure 6D). 17 of the 88 stem cell genes screened (19%) had altered expression (> 2-fold) after Lip-FLLL32 treatment, including 6 embryonic stem cell markers (*ITGB1, IFITM2, KIT, LCK, FOXD3* and *PODXL*), 2 ectoderm markers (*VIM* and *NES*), 1 trophoblast marker (*PSG3*), and *HBB, SCGB3A2, NPPA, SERPINA1, GCM1*, *GAL*, *PTEN*, and *ROXO1*. Our data suggest that Lip-FLLL32 treatment eliminates pancreatic CSCs by modulating the expression of a wide spectrum of stem cell genes as well as STAT3.

## DISCUSSION

In the current study, we found that high nuclear pSTAT3 expression correlates with advanced tumor grade of pancreatic cancer and poor patient survival. To effectively target pSTAT3, we prepared Lip-FLLL32, a liposome-formulated pSTAT3 inhibitor FLLL32, which efficiently suppressed cell growth and colony formation in pancreatic cancer cells with constitutively activated STAT3 *in vitro* and inhibited xenograft tumor growth *in vivo*, accompanied by reduction of pSTAT3 and its target genes expression. Lip-FLLL32 sensitized pancreatic cancer cells to gemcitabine chemotherapy and X-ray radiotherapy by repressing tumorsphere formulation and reducing ALDH1^+^ cell population in xenograft tumors, suggesting the reduction of CSCs. Our results support that Lip-FLLL32 could be a promising therapeutic agent sensitizing pancreatic cancer cells to chemo/radio-therapy via efficient delivery of FLLL32 and then inhibition of STAT3 *in vivo*.

**Figure 7:**
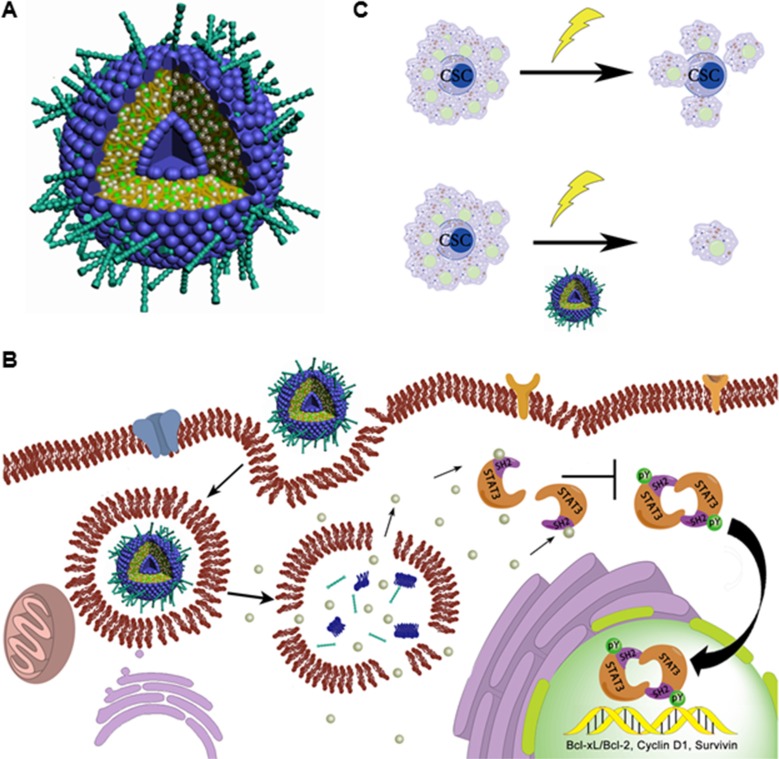
Proposed working model (**A**) Schematic representation of PEGylated liposomes encapsulating FLLL32 as spherical vesicles. FLLL32 (silver balls) and cholesterol (green balls) are encapsulated inside of the lipid bilayer formed by EPC (blue hydrophilic heads and deep yellow hydrophobic tails) with PEG coating (cyan sticks). (**B**) Schematic representation of FLLL32 delivery, intracellular releasing and affecting processes. Upon release from liposome in the cytosol, FLLL32 binds to the SH2 domain of STAT3, which inhibits the phosphorylation, dimerization and nuclear transloacation of STAT3 and subsequently transcription of STAT3 target genes. (**C**) Working model of combination therapy. Radiotherapy only eliminates proliferating cancer cells, while combination treatment results in both killing of proliferating cancer cells and eradicating of CSCs by Lip-FLLL32.

X-ray radiation is a conventional adjuvant therapy for many types of cancer including pancreatic cancer. However, resistance and recurrence frequently occur in patients receiving radiation therapy. The main mechanism for failure of radiation therapy is that radiation only kills the actively proliferating differentiated cells but not the resistant CSCs, which are reported to be responsible for tumor recurrence [43]. In this study, we show that liposomal FLLL32 not only inhibits pancreatic cancer cell growth but also sensitizes pancreatic cancer cells to radiation by attenuating the radiation-induced increase of ALDH1-positive stem cell-like population. Therefore, combination of radiation with liposomal FLLL32 can kill both proliferating cancer cells and CSCs through inhibiting pSTAT3, which will lead to sensitize pancreatic cancer to radiotherapy and overcome CSC-induced recurrence after radiotherapy (Figure 7).

Strategies for therapeutic intervention into STAT3 signaling include inhibition of STAT3 activation through upstream targets (EGFR, JAK, etc), inhibition of intermolecular interaction targeting STAT3 SH2 domain, inhibition of nuclear translocation of STAT3, and inhibition of STAT3 transcriptional activities [19]. As STAT3 sits at a convergence of multiple signaling pathways, upstream targets inhibition to block STAT3 activation just has moderate efficacy and is easy to gain resistance due to activation of alternative pathways commonly happened to STAT3 upstream target inhibitors. FLLL32 works as a potent small molecule inhibitor of pSTAT3, which binds to the SH2 domain of STAT3, and specifically inhibits STAT3 phosphorylation [20].

In this study, we made liposomal formulation of FLLL32 to improve its aqueous solubility for *in vivo* delivery. Liposomes are versatile drug delivery carriers. Delivery of hydrophobic drugs in liposome system not only solves aqueous solubility problem and allows those drugs to be administrated via i.v. injection, but also passively targets them to solid tumors leading to enhanced antitumor efficacy. We show that liposome-formulated FLLL32 is more concentrated in tumors compared to other organs including liver and spleen, two main organs involving in nonspecific clearance of nanoparticles. Liposomal FLLL32 is also more accumulated in tumors comparing to free FLLL32. Thus Lip-FLLL32 prepared here with PEG-coating and well-controlled size can efficiently deliver FLLL32 and accumulated in tumors *in vivo*.

Liposomal FLLL32 is about two-fold less efficient than free FLLL32 in short-term *in vitro* assays (cytotoxicity and reporter assays). This may be due to the slow release property of liposomes, since it takes 12 hours more to inhibit pSTAT3 and downstream genes at same amount for encapsulated FLLL32 than that of free FLLL32. As a matter of fact, liposomal FLLL32 and free FLLL32 show similar effects in long-term *in vitro* assays (colony, tumorsphere formation and clonogenic assays). The maximal tolerated dose (MTD) of FLLL32 in liposomal formulation is higher than that of FLLL32 in Cremophor formulation, demonstrating that liposomal FLLL32 is safer and less toxic *in vivo*. The MTD of Cremophor formulation is used in the *in vivo* efficacy study to compare two formulations directly. Although liposomal FLLL32 does not display obvious advantage in inhibiting tumor growth at the same dosage compared to FLLL32 in Cremophor formulation, it more efficiently delays tumor growth. Furthermore, higher MTD and better tumor/non-tumor ratios make it possible to achieve better anti-tumor efficacy at a higher dose.

Tumor-associated inflammation has been recognized as an important hallmark of cancer including pancreatic cancer [44]. It promotes tumor development and progression. Recent evidence suggests that STAT3 plays a crucial role in inducing and maintaining a pro-carcinogenic inflammatory microenvironment [11]. It is found that STAT3 forms a positive loop with two inflammatory cytokines IL-6 and IL-11 in pancreatic cancer: STAT3 directly affects the expression of IL-6 and IL-11, both of which are STAT3 activating cytokines [9, 45, 46]. So it would be attractive to investigate if Lip-FLLL32 treatment inhibits pancreatic cancer growth, at least in part, through interfering tumor microenvironment.

Lastly, we identify that Lip-FLLL32 treatment in PANC-1 cells affects expression of 17 more stem cell genes by PCR array. We will further explore Lip-FLLL32's function in CSCs. The detailed mechanisms of Lip-FLLL32 modulating those genes, dependent or independent of STAT3 inhibition, selectively in cancer or not, are still under investigation.

In conclusion, we describe the delivery of a STAT3 inhibitor, FLLL32, by liposomes (Figure 7A), which improves not only the bioavailability of FLLL32 but also the selective distribution of FLLL32 to pancreatic tumors. By targeting STAT3, Lip-FLLL32 inhibits both proliferating pancreatic cancer cells and pancreatic cancer stem cells, resulting in reversal of radioresistance induced by CSCs (Figure 7B–7C). Therefore, our data provide basis for a promising therapeutic strategy for the deadly pancreatic cancer.

## MATERIALS AND METHODS

### Cell culture and reagents

Human pancreatic cancer cell lines and normal human lung fibroblast cell line WI-38 were purchased from American Type Culture Collection and cultured in high-glucose Dulbecco's modified Eagle medium (DMEM; HyClone) supplemented with 10% fetal bovine serum (FBS; HyClone) and 1% antibiotics (HyClone) in a 5% CO_2_ humidified incubator at 37°C. The reagents cholesterol, L-α-phosphatidylcholine (Egg PC) and 1,2-distearoyl-*sn*-glycero-3-phosphoethanolamine-N-[methoxy (polyethylene glycol)-2000] (ammonium salt; DSPE-PEG-OMe) were purchased from Avanti Polar Lipids. 1,1′-Dioctadecyl-3,3,3′,3′-Tetramethylindotricarbocyanine Iodide (DiR; DiIC_18_(7)) was purchased from Life Technologies. FLLL32 and pLucTK, pLucTKS3 luciferase reporter constructs were prepared as described previously [20]. Antibodies against STAT3, Phospho-STAT3 (Tyr705), PARP, and Bcl-xL were purchased from Cell Signaling Technology, Cyclin D1 (H-295) and Bcl-2 (C-2) were purchased from Santa Cruz Biotechnology. Anti-Survivin antibody was obtained from Novus Biologicals and anti-β-Actin (AC–74) was obtained from Sigma. Primers with the following sequences for qRT-PCR were obtained from Integrated DNA technologies: *Bcl-xL* forward 5′-ATG GGG TAA ACT GGG GTC G-3′ and reverse 5′-GGC TCT AGG TGG TCA TTC AGG-3′, *CCND1* forward 5′-CCG TCC ATG CGG AAG ATC-3′ and reverse 5′-ATG GCC AGC GGG AAG AC-3′, *Survivin* forward 5′-TGC CTG GCA GCC CTT TC-3′ and reverse 5′-CCT CCA AGA AGG GCC AGT TC-3′, *18srRNA* forward 5′-GTA ACC CGT TGA ACC CCA TT-3′ and reverse 5′-CCA TCC AAT CGG TAG TAG CG-3′.

### Tissue samples

Tissues samples of 156 pairs of pancreatic cancer tissue and adjacent non-tumor tissues (within the cancer edge of 5 cm) were obtained from National Engineering Center for Biochip (NECB) in China. The following histopathological factors were evaluated: tumor histologic subtype, grade, tumor size, location, TNM stage, and pathological stage according to the 2009 American Joint Committee on Cancer (AJCC) TNM staging system (7th edition). Follow-up data was included: date of pancreatectomy, survival status, date of death, and/or date of last follow-up. This study was reviewed and approved by the Institutional Review Board of the Fourth Military Medical University, Xi'an, China.

### Immunohistochemistry analysis

TMA staining was performed by standard immunohistochemistry procedures [47]. To confirm the specificity of the primary antibodies, tissue sections were incubated in the absence of the primary antibodies and with control mouse IgG. The number of positively stained cells and the intensity of positive staining were independently scored by 2 pathologists in a blinded manner. The percentage of positive stained cells was scored as: 0, no colored cells; 1, 1–9%; 2, 10–49%; 3, 50–79%; 4, 80–100%. The intensity of positive immunostaining was classified into four categories: 0, 1, 2, and 3 representing no visible staining; light brown, mid-brown and dark brown staining, respectively, with the same intensity covering more than 75% of the staining area. The immunostaining of each tissue was assessed in 5 areas of the acquired images of each tissue section and the mean of these 5 scores was calculated. The total immunostaining score was calculated by multiplying intensity score by positivity score, and categorized as four expression subgroups: no, negative total score; weak, total score 1–4; moderate, total score 5–8; intense, total score 9–12. For statistical analysis, the stained tumor tissues were divided into two groups: the low-expression group (score ≤ 4, no to weak) and the high-expression group (score > 4, moderate to intense).

### Preparation of Liposome-encapsulated FLLL32 (Lip-FLLL32)

Liposomal FLLL32 was prepared by dissolving co-lipids (cholesterol, Egg PC and DSPE-PEG-OMe) and FLLL32 in chloroform at a 2:18:1:2 ratio in a glass vial. The organic solvent was removed by vacuum and the remaining dried film was then kept under high vacuum overnight. PBS (*in vitro*) or 5% dextrose (*in vivo*) was added to the vacuum-dried lipid film and the mixture was allowed to hydrate for 30 min by shaken in a 45°C water bath to generate multilamellar vesicles. Small unilamellar vesicles were then prepared by sonication of the multilamellar vesicles in an ice bath for 20–30 min until clarity, using a probe sonicator at 50% duty cycle. Empty liposome (Lip only) was prepared using the same procedure without FLLL32, while liposomal DiR was prepared by dissolving co-lipids (cholesterol, Egg PC and DSPE-PEG-OMe), FLLL32 and DiR in chloroform at a 2:18:1:2:0.2 ratio. The particle size and zeta potential of prepared liposome were determined by Malvern Zetasizer Nano-zs90.

### Scanning transmission electron microscopy (STEM)

6 μL of Lip only or Lip-FLLL32 (1:1 dilution by nano water) was dropped to the grid and wicked off immediately (kim wipe). The grid was let dry for 3 min following by adding 10 μL 4% of uranyl acetate (UA) in DI water and incubating for 5 min. UA was then wicked off and the grid was let dry for another 1 min and put in grid holder until ready to view under FEI Tecnai G2 Polara 200 kV TEM.

### Tumorsphere culture

FLLL32 or Lip-FLLL32 treated cells were suspended in DMEM/F12 serum-free medium containing 1% N2, 2% B27, 1% antibotic-antimycotic (Invitrogen), 20 ng/ml human FGF-2 (Sigma), and 100 ng/ml EGF (Invitrogen), and were plated in 24-well ultra-low attachment plates (Corning) with 2,000 cells per well. Ten to14 days later, spheres in plates were quantified using an inverted microscope (Olympus) at 100×, 200×, and 400× magnifications.

### Animal model: *in vivo* analysis of MTD, biodistribution and therapeutic efficacy

Female athymicNCr-nu/nu mice of 5–6 weeks old were used for maximal tolerated dose (MTD) study. Three mice in each group were administrated with gradually increased doses of FLLL32 (10 mg/kg, 15 mg/kg and 22.5 mg/kg) in liposome or Cremophor formulation via tail i.v. injection every other day. Mice status and body weight were monitored every day. The dose that causes mice with body weight decreased more than 20% is considered intolerable. For the following experiments, each mice was inoculated subcutaneously on both flanks with 5 × 10^6^ cells in 0.2 mL DMEM. Tumor sizes were measured using a caliper twice a week. Tumor volume was calculated using the formula: (length × width^2^)/2, as we described previously [48]. In biodistribution study, three mice with tumor size around 200 mm^3^ were used: one mouse was administrated with 200 μL DiR in 10% ethanol through tail i.v. injection, the other two were administrated with 200 μL liposomal DiR and FLLL32, and the concentration of DiR in both formulations was 0.1 mg/ml. At scheduled time points, mice were anesthetized and scanned using a Carestream Molecular Imaging System, *in vivo* Multispectral FX PRO, with the excitation at 750 nm and the emission at 830 nm using a 30s exposure time. X-ray images were also taken for tumor location and over laid with corresponding near-infrared (NIR) images. 96 h after injection, the mice were euthanized by CO_2_ overdose, main organs and tumors were isolated and taken NIR and X-ray imaging. In efficacy study, the mice with tumors around 100 mm^3^ were randomized to 3 or 4 groups with 6 mice each group. In monotherapy study, mice were administrated with 15 mg/kg FLLL32 or Lip-FLLL32 by tail i.v. injection three times per week for three weeks, or left untreated as a control group. Free FLLL32 was formulated in a vehicle composed of a 1:1 blend of Cremophor^®^ EL (polyethoxylated castor oil) and ethanol, which was diluted 5-fold by 5% dextrose solution before administration. In combination therapy, mice were received with Lip only, 2 Gy X-ray radiation, 15 mg/kg Lip-FLLL32 or 2 Gy X-ray radiation plus 15 mg/kg Lip-FLLL32 three times per week for three weeks, respectively. The tumor sizes and animal body weights were measured twice a week. After one-week treatment, one mouse from each group was sacrificed and tumors were dissected for western blot, PCR or ALDH1 staining. At the end of treatment, one tumor from each group was collected for histologic analysis including hematoxylin and eosin (H & E) staining, terminal deoxynucleotidyl transferase biotin-dUTP nick end labeling (TUNEL) staining using an *in situ* ApopTag kit for apoptosis detection, and anti-mouse CD31 immunostaining for tumor blood vessels, as we described previously [33]. All animal experiments were done according to the protocol approved by the University of Michigan Guidelines for Use and Care of Animals.

### ALDEFLUOR assay

The ALDEFLUOR kit (Stem Cell Technologies) was applied to analyze the population with ALDH enzymatic activity using a FACS Calibur, as previously described [49]. Briefly, cells were incubated in ALDEFLUOR assay buffer containing ALDH substrate BAAA (1.5 μM per 1 × 10^6^ cells) at 37°C for 45 min. In each experiment, cells were also incubated under identical conditions with negative control, 15 μM of ALDH inhibitor diethylaminobenzaldehyde. Cells with ALDH activity convert BAAA to the negatively charged fluorescent product, BAA, which can be measured fluorescence on a flow cytometer. To eliminate cells of mouse origin from the xeno-transplanted tumors, cells were firstly stained with an anti-H_2_Kd antibody (diluted 1:200, 20 min on ice; BD Biosciences) followed by staining with a secondary antibody labeled with PE (diluted 1:250, 20 min on ice; Jackson Labs) and then were subsequent to ALDEFLUOR assay.

### Stem cell PCR array

Total RNAs isolated from PANC-1 cells treated with Lip-FLLL32 or Lip only were reverse transcribed into complementary DNA, and amplified by Human Stem Cell Primer Library (HSCL-I) from RealTime Primers LLC (Elkins Park), which contains 88 primer sets directed against stem cell-related genes and 8 housekeeping gene primer sets as we described previously [17].

### Statistical analysis

Student *t*-test, one-way and two-way ANOVA were employed to analyze the *in vitro* and *in vivo* data using Prism 5.0 software (GraphPad). Categorical variables were compared using the x^2^ test. The Kaplan–Meier method and the log-rank test were used to compare overall survival, defined as the time of patients from surgery until death (patients alive were censored at the time of their last follow-up), and were conducted with statistical analysis software program SPSS 13.0 software (IBM). *P < 0.05, **P < 0.01, ***P < 0.001, as compared with the control group.

## SUPPLEMENTARY MATERIAL FIGURES AND TABLES


