# Supplementary material for: Overcoming chemo/radio-resistance of pancreatic cancer by inhibiting STAT3 signaling
Source: Oncotarget. 2016 Feb 12;7(10):11708–23. doi: 10.18632/oncotarget.7336 (PMC4905505; doi:10.18632/oncotarget.7336)
Supplement: Supplementary file 1 [file oncotarget-07-11708-s001.pdf]

Overcoming chemo/radio-resistance of pancreatic cancer by inhibiting STAT3 signaling

Supplementary Materials

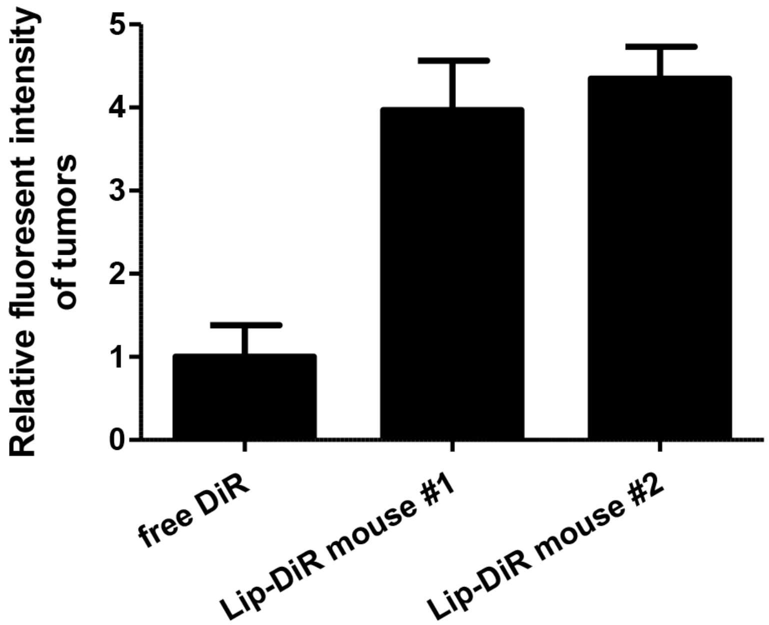

Supplementary Figure S1: Relative fluorescence intensity in tumors received free DiR or liposomal DiR, DiR signal is accumulated to about four folds in tumors given Liposome formulation comparing to free DiR.

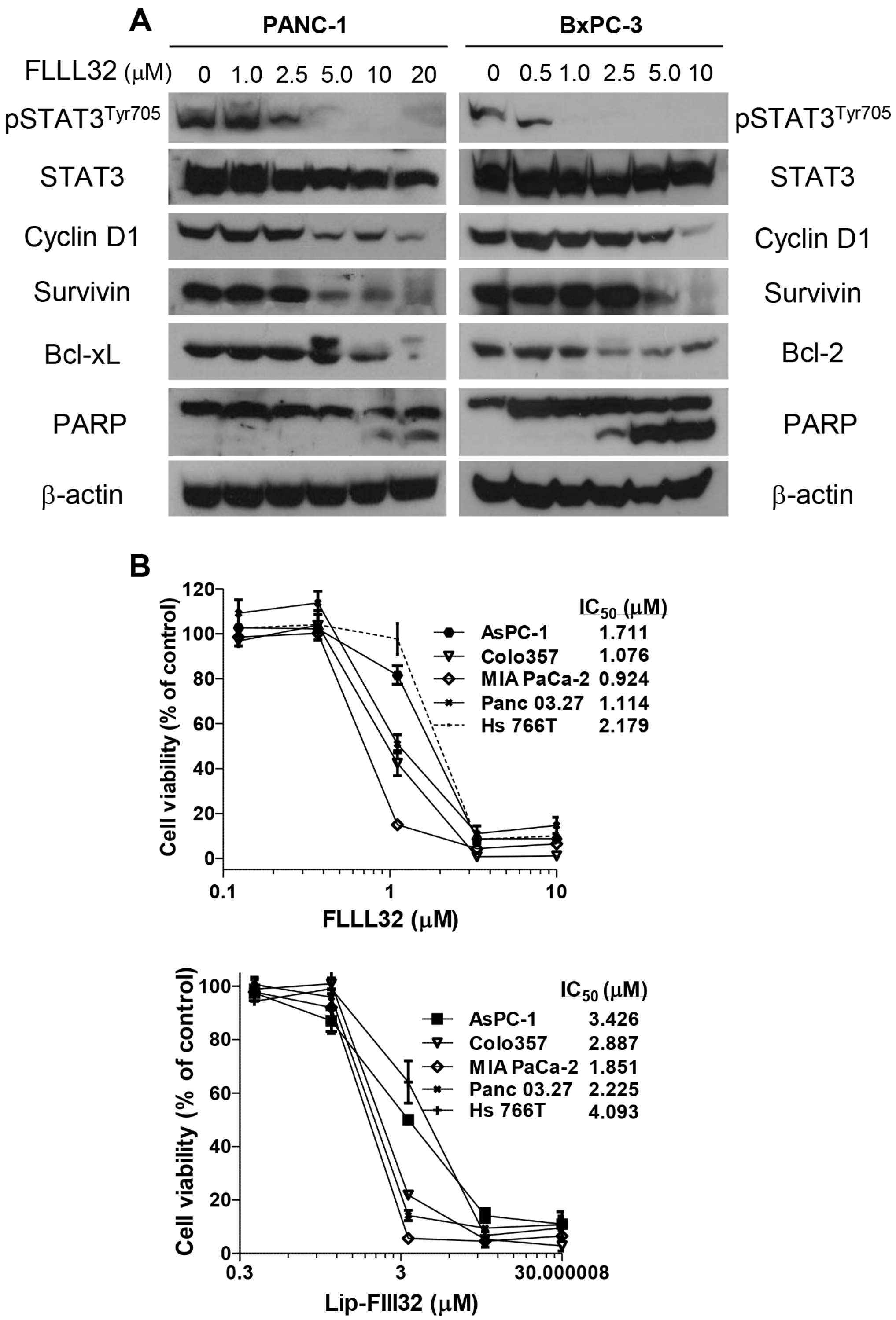

Supplementary Figure S2: (**A**) Western blot analysis of STAT3 and its target proteins in FLLL32 treated PANC-1 (left) and BxPC-3 cells (right) at different doses for 24 hours. FLLL32 dose dependently inhibits pSTAT3 and down-stream proteins. (**B**). The MTT-based cytotoxicity analysis of FLLL32 and Lip-FLLL32 against a panel of pancreatic cell lines.

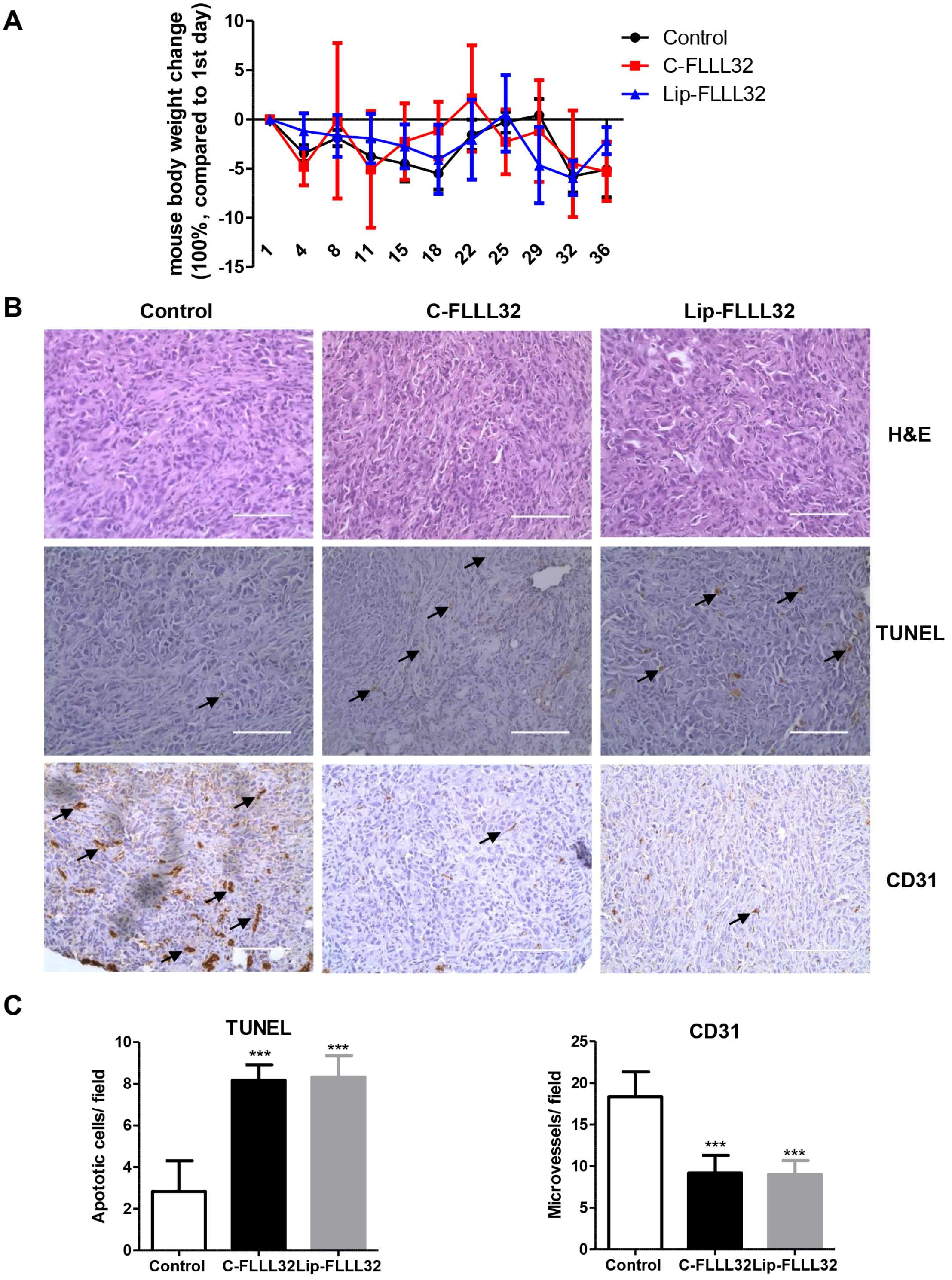

Supplementary Figure S3: (**A**) Average body weight change of mice receiving 15 mg/kg Cremophoror formulated FLLL32 or 15 mg/kg liposome formulated FLLL32, or left untreated during experimental duration. (**B**–**C**) Immunohistologic analysis of tumor tissues. One tumor from each group was taken at the end of treatment, fixed with 4% formaldehyde and processed for H & E staining, TUNEL staining and CD31 immunostaining. (B) Representative pictures (Bar: 100 μm. Magnification: 400X). In TUNEL assay, apoptotic cells were stained with brown nuclei (arrows). In CD31 staining, tumor blood vessels were stained brown (arrows). (C) TUNEL-positive cells and CD31-positive endothelial cells were quantified by randomly counting six fields. Values are mean ± SD ($n = 6$, ***$P < 0.001$, one-way ANOVA).

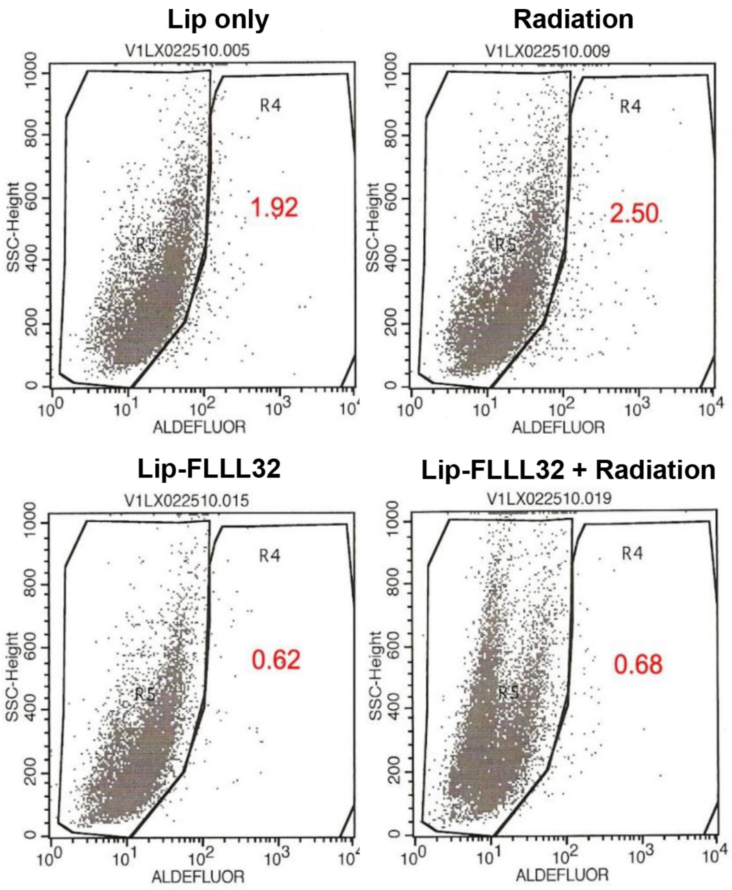

Supplementary Figure S4: Representative diagrams of ALDEFLUOR assay by flow cytometry analysis of ALDH1^{+} population in xenograft tumors from mice treated with Lip only, radiation, liposomal FLLL32, or their combination for one week as indicated in Figure 5C.

Supplementary Table S1: Correlation between nuclear pSTAT3 expression and the clinic-pathologic factors

		Low expression (score < = 4)	High expression (score > 4)	*P*
Age (*n* = 155)	≤ 60 (y)	43	45	0.816
	> 60 (y)	34	33	
Gender (*n* = 156)	Male	43	55	0.075
	Female	34	24	
Tumor type (*n* = 155)	Duct adenocarcinoma	76	63	**0.037**
	Glandular cancer	6	15	
Tumor size (*n* = 156)	≤ 3 (cm)	25	52	0.695
	> 3 (cm)	28	51	
Tumor grade (*n* = 149)	I + II	65	48	**0.011**
	III	12	24	
Tumor location (*n* = 146)	Head of pancreas	50	55	0.236
	Body/Tail of pancreas	24	17	
T stage (*n* = 155)	T1 + T2	61	67	0.456
	T3	15	12	
N stage (*n* = 141)	0	36	42	0.718
	≥ 1	31	32	
AJCC stage (*n* = 156)	1	29	35	0.399
	2 + 3	48	44	
Patient survival (*n* = 79)	Live	15	8	0.169
	Dead	27	29	
Smoking (*n* = 142)	no	44	45	0.632
	yes	24	29	
Drinking (*n* = 140)	no	46	49	0.846
	yes	21	24	

Supplementary Table S2: Maximal tolerated dose study

Dose (mg/kg)	Mice status	
	Cremophor ® EL and ethanol	Liposome
10	Good	Good
15	Good	Good
22.5	Bad	Good
